# Full-length 16S rRNA nanopore sequencing enables species resolution of *Fusobacterium* associated with colorectal cancer

**DOI:** 10.1080/19490976.2026.2656004

**Published:** 2026-04-10

**Authors:** William Rosenbaum, Marina Rubio Garcia, Anna Löfgren-Burström, Pär Larsson, Sofia Edin, Vicky Bronnec, Richard Palmqvist

**Affiliations:** a Department of Medical Biosciences, Pathology, Umeå University, Umeå, Sweden

**Keywords:** *Fusobacterium* species identification, colorectal cancer, full-length 16S rRNA, oxford nanopore sequencing, gut microbiota profiling, pathobiont

## Abstract

Recent studies have revealed that the long-recognized link between the historically defined *Fusobacterium nucleatum* group and colorectal cancer is largely driven by *Fusobacterium animalis*. This species, along with two others (*Fusobacterium polymorphum* and *Fusobacterium vincentii*), was recently reclassified as distinct from *F. nucleatum*, highlighting functional divergence within this group. Due to their close genetic relatedness, traditional partial 16S rRNA gene sequencing lacks the resolution to reliably distinguish these species. Nevertheless, accurate species-level identification remains essential in cancer-associated microbiome research. Here, we demonstrate that full-length 16S rRNA sequencing using Oxford Nanopore Technology, combined with a novel custom demultiplexing software, enables robust species-level discrimination within the *Fusobacterium* genus. Our approach accurately classified clinically relevant *Fusobacterium* species and recovered their expected proportions from whole cells, DNA mixtures, and clinical CRC specimens. This method provides high-resolution profiling to elucidate species-specific roles of *Fusobacterium* in colorectal cancer.

## Graphical abstract

**Figure:**
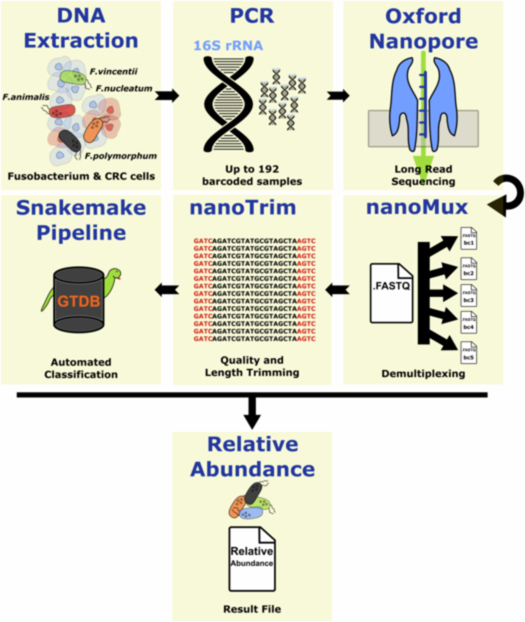

Schematic representation of the workflow outlined in this article. DNA is extracted and subjected to PCR amplification of the full-length 16S rRNA gene. The samples are multiplexed and sequenced on a Nanopore flow cell. The sequences are demultiplexed using nanoMux (introduced in this article), and the reads are trimmed and analyzed using the custom Snakemake pipeline described in this article.

## Introduction

Colorectal cancer (CRC) is one of the most common forms of cancer globally, with over 1.9 million new cases and almost 1 million deaths in 2022.^1‐3^ CRC is a multifactorial disease, caused by both genetic and environmental factors.^4^^,^^5^ Increasing evidence suggests that the microbiome plays a crucial role in the development and progression of CRC.^5‐10^ Interestingly, the presence of bacteria within the tumor tissue itself, *i.e.* intratumoral bacteria, appears to affect tumor biology and may modulate the response to therapy, such as immunotherapy.^11^^,^^12^ In tumor tissue from CRC patients, bacteria originating from the commensal oral flora are enriched compared to tissue from healthy controls.^9^^,^^13^^,^^14^ One of the well-studied bacteria in this context is *Fusobacterium nucleatum*, a Gram-negative, obligate anaerobic and biofilm-associated bacterium.^9,15‐17^

**Figure 1.**
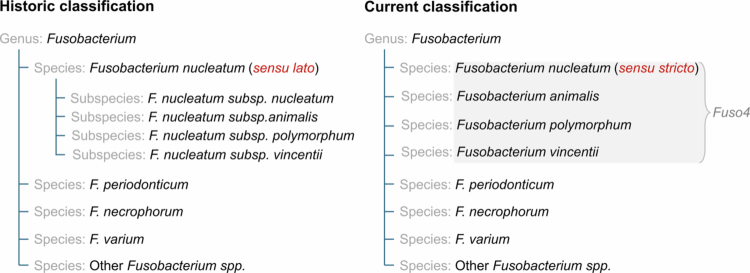
Conceptual comparison of historical and current taxonomic frameworks of *Fusobacterium* species. Under the historical classification (*sensu lato*), multiple distinct lineages were grouped as subspecies within the broadly defined *F. nucleatum* species complex. In the current classification (*sensu stricto*), these same lineages are now recognized as separate species within the genus *Fusobacterium*, with *F. nucleatum* restricted to a single, well-defined lineage. The underlying phylogenetic relationships between these lineages remain unchanged; only their taxonomic rank and nomenclature have been revised to better reflect evolutionary distances. Three additional *Fusobacterium* species included in this study are also shown and the non-exhaustive group is referred to as “other *Fusobacterium* spp.” This study uses the collective term “The four *Fusobacterium*” to refer to the four historical subspecies, abbreviated as “*Fuso4*”.

The *F. nucleatum* group (*sensu lato*) has traditionally been classified into four subspecies, namely *F. nucleatum* subsp. *nucleatum*, *F. nucleatum* subsp. *animalis*, *F. nucleatum* subsp. *vincentii* and *F. nucleatum* subsp. *polymorphum*. Recent genomic analyzes have suggested elevating the four subspecies into separate species, namely: *Fusobacterium nucleatum sensu stricto* (historically *F. nucleatum* subsp. *nucleatum*), *Fusobacterium animalis*, *Fusobacterium vincentii*, and *Fusobacterium polymorphum.*^15‐21^ Previous studies reported the *F. nucleatum* group to be associated with CRC without distinguishing between its subspecies. Using the novel taxonomy, recent studies have highlighted that the enrichment in CRC tissues is skewed towards *F. animalis.*^9^^,^^16^^,^^22^ The present study is aligned with the updated taxonomic nomenclature (Figure 1).

To accurately study how gut microbiota composition and specific bacterial species affect the development and progression of CRC, bacterial classification is essential. Conventionally, only parts of the bacterial 16S rRNA gene (e.g., hypervariable regions V3–V4) are amplified by PCR and subsequently sequenced with technologies producing short reads.^23^ These short-read technologies, e.g., Illumina-based sequencers, have limitations.^24^ Indeed, by targeting only portions of the 1500 base pair (bp) of the 16S rRNA gene, the resulting classification lacks the precision achieved by sequencing the entire gene. Ideally, classification at the species level requires sequencing the whole 16S rRNA gene.^25^ Technologies that produce long reads, such as Oxford Nanopore Technology (ONT), preserve the entire information of the 16S rRNA gene, thus enabling higher resolution classification.^26^ Due to the limitations of utilizing partial 16S rRNA sequencing for bacterial classification, using 16S rRNA as a marker is regarded as insufficient for reliable species-level resolution, especially within closely related species, such as the historic *Fusobacterium nucleatum* group.^10^^,^^27^ Moreover, the accuracy of 16S rRNA-based taxonomic classification depends heavily on the reference database used for sequence annotation.^28^

In this study, we evaluate the capability of full-length 16S rRNA sequencing, utilizing the latest Oxford Nanopore chemistry and flow cells, for accurate species delineation of *F. nucleatum*, *F. animalis*, *F. vincentii*, and *F. polymorphum*. Experimental validation in this manuscript was conducted for a total of seven *Fusobacterium* species through sequencing across various sample types. *In silico* analyses further suggest that this approach could be extended to the entire genus, although additional experimental validation remains necessary. To substantially reduce sequencing costs, we provide a custom demultiplexing software and an optimized PCR protocol that enables up to 192 samples to be sequenced on a single flow-cell, along with a publicly available bioinformatic pipeline for data processing.

## Results

### Demultiplexing samples with unique dual index barcodes

We developed nanoMux (part of the nanoSweet tool suite; https://github.com/willros/nanoSweet) to efficiently demultiplex pooled samples sequenced on a single flow cell using unique dual index barcodes. This demultiplexing approach had not been previously implemented in existing basecalling software, necessitating custom development. For each read, the algorithm extracts the first *p* nucleotides (5’ end) and the last *p* nucleotides (3’ end), comparing these regions against expected barcodes using Levenshtein distance. A read is assigned to a sample only when both the forward barcode at the 5’ end and the reverse complement of the reverse barcode at the 3’ end match within a mismatch threshold (*k*). This dual-match requirement ensures high specificity, as reads must contain both expected barcodes in the correct orientation to be assigned to a sample.

Our approach leveraged the combinatorial nature of unique dual index barcodes, pairing the same forward barcode with multiple reverse barcodes to generate numerous unique sample identities from a limited barcode set. Using 8 forward barcodes combined with 24 reverse barcodes, we achieved 192 unique dual index barcode combinations without requiring individual barcodes per sample. nanoMux was implemented in pure C with no external dependencies, enabling fast, lightweight demultiplexing directly on basecalled FASTQ files.

We applied the following default parameters: *k* = 3 (maximum 3 mismatches in Levenshtein distance) and *p* = 300 bp for the search window. The *k* = 3 threshold successfully tolerated Nanopore sequencing errors while maintaining barcode specificity and minimizing misclassification between samples (Figure S1). The *p* = 300 bp window accommodated positional variability in barcodes and adapters due to truncation and terminal errors. These parameters balanced sensitivity and specificity for reliable demultiplexing across sequencing variability, and our implementation successfully demultiplexed pooled samples with high accuracy.

### Analysis of 16S rRNA PCR, sequencing and classification pipeline reveals on-target amplification and stability across runs

**Figure 2.**
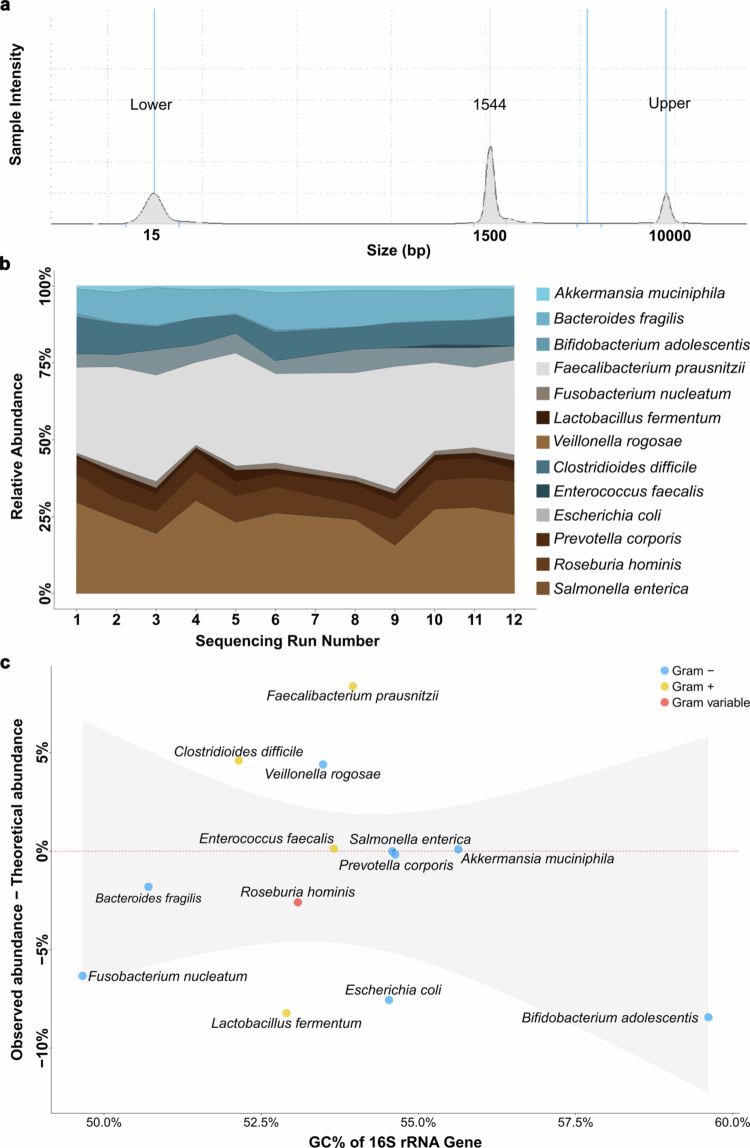
PCR and sequencing validation of bacterial detection in a host–bacteria mock community**.** (a) Tapestation electropherogram of one representative sample after PCR amplification, using dual barcoded primers and the Platinum SuperFi II polymerase. One distinct peak at 1500 bp, which corresponds to the expected length of the full-length 16S rRNA gene. (b) Sequencing of the host–bacteria mock community. Each sequencing run stems from a distinct PCR reaction, and each sample was sequenced on separate flow cells. The species are presented according to the NCBI nomenclature. (c) Comparison of deviations from theoretical and observed abundance, as a function of GC%. The Y axis denotes the difference between the observed and theoretical abundance for species in the mock community, and the X axis shows the mean GC% content in the 16S rRNA gene for the species in the mock community, calculated from GTDB.

To create a realistic host–bacteria suspension, the ZymoBIOMICS Gut Microbiome Standard was mixed with human RKO cells at a ratio of 1:10. The ZymoBIOMICS Gut Microbiome Standard contains a mixture of 18 bacterial strains (including 5 different strains of *E. coli*, collapsed under *E. coli* in the present study), 2 fungal strains and 1 archaeal strain. To test the consistency and potential bias of the method, we sequenced the host–bacteria mock community in 12 independent runs, each originating from a separate PCR amplification. The 16S rRNA gene was amplified for 30 PCR cycles using the Platinum SuperFi II polymerase, as it showed higher amplification efficiency than another polymerase tested (data not shown). To evaluate the efficiency and potential off-target products that may be introduced by PCR of the 16S rRNA gene, we inspected the amplicons generated from each reaction on the Agilent Tapestation platform. A distinct peak at the expected size of approximately 1500 bp was observed (Figure 2a). Next, the PCR products were sequenced on different flow cells (Figure 2b). The relative abundance profiles of individual bacterial species were highly consistent across all sequencing runs (Figure 2b). To eliminate off-target species, we filtered the resulting classification table to only contain species found in the ZymoBIOMICS Gut Microbiome Standard. Each member of the mock–host community was accurately classified to the species level (Supplementary Data 1; Supplementary Data 2). Mean quality scores of the sequences can be found in Figure S2. The ZymoBIOMICS Gut Microbiome Standard contains bacteria with very low theoretical relative abundance of the 16S rRNA gene, including *Methanobrevibacter smithii* (0.066%), *Salmonella enterica* (0.009%), *Enterococcus faecalis* (0.0009%) and *Clostridium perfringens* (0.0002%). Owing to their low representation, no sequencing reads were assigned to these taxa and were therefore not present in the resulting relative abundance table (Supplementary Data 2). Collectively, these results confirm that our method is stable across runs and produces comparable and reliable results over time.

To assess whether the GC-content of the 16S rRNA genes and Gram-stain status could influence the observed abundance of species in the host–bacteria mock community, and whether those factors should be used for normalization, we calculated the GC-content for each *Fusobacterium* species using sequences from the Genome Taxonomy Database (GTDB) and Gram-stain information from the ZymoBIOMICS Gut Microbiome Standard protocol sheet. The association between theoretical and observed deviation and GC% content was tested with a linear model controlling for Gram stain (Figure 2c). No significant associations were found, indicating neither GC-content nor Gram-stain status affects the outcome.

### Full-length 16S rRNA comparative genomics distinguishes the four *Fusobacterium* species

To assess the theoretical ability to differentiate *Fusobacterium* species, genetic similarities among their 16S rRNA gene sequences were calculated using reference sequences from GTDB. Using only full-length, complete and unique 16S rRNA *Fusobacterium* sequences from GTDB, we analyzed 7, 54, 31, and 34 16S rRNA gene sequences for *F. nucleatum*, *F. polymorphum*, *F. vincentii*, and *F. animalis*, respectively (Supplementary Data 3).

**Figure 3.**
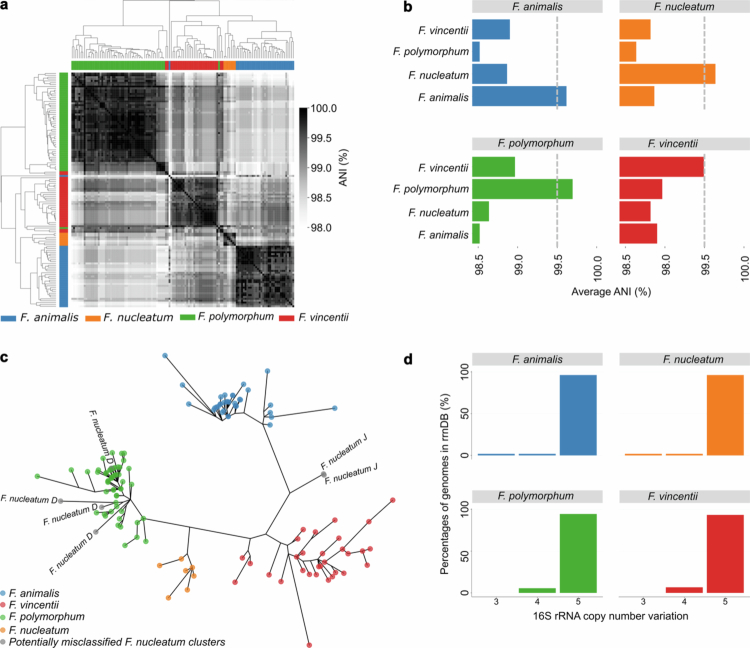
16S rRNA gene sequence comparison among *Fusobacterium* species historically classified within the *Fusobacterium nucleatum* group (*sensu lato*). (a) Heatmap showing ANI values calculated with skani from the 16S rRNA gene sequences, organized into groups using unsupervised clustering in Seaborn. (b) Mean ANI values calculated for the four *Fusobacterium* species, with the red line indicating the 99.5% ANI threshold. (c) Unrooted phylogenetic tree of the four *Fusobacterium* species based on the 16S rRNA gene sequence, visualized using ggtree in R. (d) Histogram of CNV for the four *Fusobacterium* species calculated from rrnDB. Panel (c) was generated based on the original GTDB classification, including clusters J and D historically classified as *Fusobacterium nucleatum* but likely misclassified).^21^ All other analyses were conducted after removing sequences labelled as clusters “J” and “D” from the reference database.

Genetic similarity of the 16S rRNA genes based on Average Nucleotide Identity (ANI), used here to quantify pairwise 16S rRNA gene similarity rather than for whole-genome species delineation, was conducted using skani triangle, which calculates pairwise ANI values for all sequences to generate a complete similarity matrix. To visualize the subsequent similarity matrix, the clustermap function from Seaborn was used, which performs unsupervised clustering using the average linkage method and Euclidean distance metric (Figure 3a). Notably, despite high overall ANI similarity, sequences clustered distinctly by species, indicating that the 16S rRNA gene is sufficient for resolution at the species level.

To provide a summarized overview of the ANI relationships, the mean ANI value for each species was calculated relative to every other species (Figure 3b). The findings highlight that an ANI cut-off of approximately 99.5% is sufficient for accurate species classification across all species (Figure 3b). An ANI cut-off of 99.5% corresponds to approximately 7–8 polymorphic sites between species, given that the 16S rRNA gene is about 1500 bp in length.

To further assess the theoretical feasibility of delineation, we constructed phylogenetic trees using MUSCLE alignments and IQ-TREE for the 16S rRNA sequences of the four species (Figure 3c). This analysis corroborated the conclusions from the ANI analysis. Once again, the four *Fusobacterium* species formed distinct clades, bringing additional evidence that the full-length 16S rRNA gene indeed can function as a taxonomic marker. An extended analysis across the entire genus further suggests that the full-length 16S rRNA gene has the potential to achieve complete species-level discrimination (Figure S3). To provide information usable in downstream analysis, we also calculated copy number variations (CNV) frequencies for the four species, based on the Ribosomal RNA Operon Copy Number Database (rrnDB). Notably, all species exhibited almost identical CNV values (Figure 3d).

During the initial analysis, a subset of sequences classified as *F. nucleatum* in the GTDB database did not cluster with the main *F. nucleatum* group (labeled as clusters “J” and “D” in Figure 3c). Closer examination revealed that these sequences ambiguous taxonomic annotations, consistent with the taxonomic inconsistencies reported by Zepeda-Rivera et al.^21^. Phylogenetic analyses were first performed using the original GTDB classifications, including the uncertainly labeled “J” and “D” clusters (Figure 3c). However, these sequences were excluded from all downstream analyses. Together, these findings support the hypothesis that species within the *Fusobacterium nucleatum sensu lato* group can be reliably distinguished using the full-length 16S rRNA gene as a species-level marker.

### 16S rRNA sequencing of *Fusobacterium* cultures enables clear delineation of different species

With the theoretical potential of distinguishing between *Fusobacterium* species based on full-length 16S rRNA sequences, we aimed to explore if these findings would transfer to a real-world example. To evaluate the ability of our 16S full-length sequencing pipeline to classify species, we extracted DNA from the four *Fusobacterium* species, in addition to *F. periodonticum*, *F. necrophorum* and *F. varium* (closely related to the four other species but never classified under *F. nucleatum sensu lato*, Figure 1). The 16S rRNA gene was amplified via PCR for each species and sequenced until sufficient reads per sample were reached. All isolates were processed in duplicates, and the results were filtered to only contain species with a relative abundance  >  1% (Table 1). The full list of all species detected is presented in Supplementary Data 4.

**Table 1.** Taxonomic classification of the sequencing reads obtained from pure cultures of ***Fusobacterium*** **species.** Total genomic DNA was extracted from each pure culture of reference strains followed by PCR amplification of the 16S rRNA gene prior to sequencing. Each sample was sequenced into two technical replicates.

Bacterial species (pure culture)	Replicate	Total Reads	Classified species	Reads supporting classification	
				(*n*)	(%)
*F. nucleatum*	1	69875	*F. nucleatum*	69219	99.061
	2	68020	*F. nucleatum*	67370	99.044
*F. animalis*	1	69328	*F. animalis*	69312	99.977
	2	69476	*F. animalis*	69461	99.978
*F. vincentii*	1	59876	*F. vincentii*	59865	99.982
	2	58485	*F. vincentii*	58473	99.979
*F. polymorphum*	1	67284	*F. polymorphum*	67258	99.961
	2	59741	*F. polymorphum*	59714	99.955
*F. necrophorum*	1	56607	*F. necrophorum*	56600	99.988
	2	52340	*F. necrophorum*	52337	99.994
*F. periodonticum*	1	40219	*F. pseudoperiodonticum*	38368	95.398
			*F. periodonticum*	1822	4.530
	2	39653	*F. pseudoperiodonticum*	37895	95.567
			*F. periodonticum*	1732	4.368
*F. varium*	1	49055	*F. varium*	49031	99.951
	2	49984	*F. varium*	49968	99.968

Strikingly, all sequenced *Fusobacterium* isolates were correctly classified with a sensitivity of >99%, except for *F. periodonticum* which was classified as *F. pseudoperiodonticum* around 95% of the time (Table 1). This is consistent with the very high phylogenetic similarity between *F. periodonticum* and *F. pseudoperiodonticum.*^20^ The 16S rRNA gene sequences of these two species share >99.5% identity, placing them at the boundary of 16S-based species resolution. This finding highlights a known limitation of 16S rRNA sequencing: species pairs with near-identical 16S sequences may remain difficult to resolve regardless of read length, and complementary markers (e.g., *rpoB*) may be needed in such cases. Importantly, this limitation does not affect the four species within the *F. nucleatum sensu lato* group, which are the primary focus of this study. Overall, the results show that the method is clearly able to distinguish between the target *Fusobacterium* species when applied to isolates.

**Figure 4.**
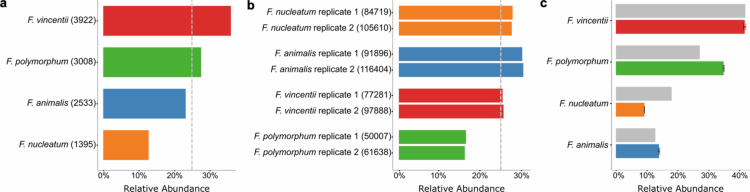
Species-level classification of *Fusobacterium* across experimental conditions. (a) Relative abundance of classified reads from pure cultures of seven *Fusobacterium* species, each sequenced in two technical replicates. (b) Relative abundance of the four *Fusobacterium* species (*Fuso4*) in a mixed whole-cell suspension pooled at a 1:1:1:1 CFU-based ratio. (c) Relative abundance of the four *Fusobacterium* species in a pre-extracted DNA mixture. The dotted line in (a) and (b) and the gray bars in (c) show the theoretical proportion for each species.

Next, we sought to evaluate our method’s ability to distinguish between *Fusobacterium* species within the same suspension (mix containing each individual species at a CFU-based ratio of 1:1:1:1; Supplementary Table 1). The results from the *Fusobacterium* suspension sequencing demonstrated that all four species were detected with only a small proportion of misclassifications (Figure 4a; Supplementary Data 5). It was anticipated that each species would constitute an equal proportion (25% for each species). However, *F. polymorphum* was detected at a lower level than the other species, while *F. nucleatum*, *F. animalis* and *F. vincentii* were slightly above the theoretical abundance. One possible explanation for this revolves around the compositional nature of the data. Since a change in the abundance of one species alters the relative proportions of the others, a shift in one abundance will inevitably affect the rest. Therefore, technical issues, including CFU miscalculation, pipetting inaccuracies during suspension preparation, or DNA extraction variability, may significantly affect the results. The most common misclassified species in both replicates was *F. canifelinum*, although detected at very low levels (Supplementary Data 5). *F. varium* and *F. necrophorum* were also detected at low levels. As these bacteria were amplified in the same PCR and sequenced on the same flow cell, the signal likely reflects barcode jumping, rather than read misclassification. Overall, the results show that the method can detect and distinguish all *Fusobacterium* species, even when present within the same sample.

We then focused on the capacity of the method to detect *F. nucleatum*, *F. animalis*, *F. vincentii* and *F. polymorphum* in a more realistic sample. We mixed the four species in CFU amounts and prepared a human cell-*Fusobacterium* suspension with a ratio of 10:1 human to bacterial cells (Supplementary Table 1). Most reads were classified as *Fusobacterium* species, with only a few reads assigned to other taxa. Both *Cutibacterium acnes* and *Acinetobacter radioresistens* (Figure 4b, Supplementary Data 5) are part of the human skin flora or ubiquitous in the environment and related to cross-contamination,^30^^,^^31^ which plausibly explains the detection of these species in this experiment.

Lastly, to eliminate potential DNA extraction bias, we generated a suspension containing defined proportions of pre-extracted DNA from the four *Fusobacterium* species individually (Supplementary Table 1). Once more, the results were stable and accurately reflected the theoretical proportions across all three replicates (Figure 4c). *F. nucleatum* was underrepresented, and *F. polymorphum* was overrepresented in contrast to the theoretical proportion. However, this is not likely an effect of differences in CNV of 16S rRNA copy number, as the CNV distributions are highly similar across the four species (Figure 3d). Plausibly, the observed deviance may also be explained by a compositional skew as described above.

In summary, the results clearly indicate the method’s capacity to delineate *Fusobacterium* species across diverse sample types, including mixed bacterial communities, suspensions with human background and pre-extracted DNA mixtures.

#### Detection of *Fusobacterium* species in clinical colorectal cancer specimens identifies *F. animalis* as the predominant species

To validate the method on clinical samples, we analyzed 20 colorectal cancer tissues paired with adjacent non-malignant tissues from the same patients. We applied a detection threshold of ≥5 classified reads per *Fusobacterium* species to define positive samples. This threshold was chosen to exceed the sporadic background signal observed in our validation experiments, where off-target *Fusobacterium* species in pure culture controls were consistently detected at fewer than 5 reads (Supplementary Data 4).

**Figure 5.**
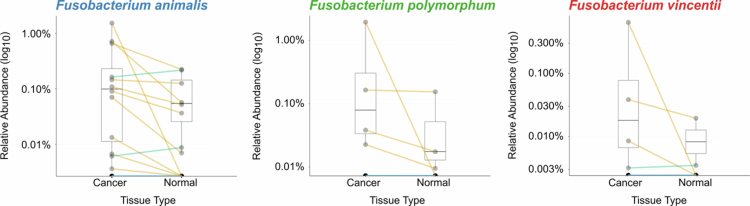
Validation cohort analysis of *Fusobacterium* species abundance in paired colorectal cancer and adjacent normal tissues. Relative abundance (log_10_ scale) of *F. animalis*, *F. polymorphum* and *F. vincentii* in paired cancer and adjacent normal tissue from the clinical CRC cohort (*n* = 20). Each line represents the direction of relative abundance change between cancer and normal tissue within each patient. Yellow represents a higher relative abundance in cancer tissue and green represents a lower abundance in cancer tissue.

Among the four *Fusobacterium* species examined, *F. nucleatum* was not detected above this threshold in any sample. *F. animalis* showed the most differential distribution (Figure 5), detected in 60% (12/20) of cancer tissues with a mean relative abundance of 0.17% compared to 40% (8/20) of normal tissues with a mean relative abundance of 0.036%. This ~ 5-fold enrichment in cancer tissue suggests potential biological relevance in colorectal cancer, consistent with recent findings.^9^ A paired Wilcoxon signed-rank test confirmed that *F. animalis* abundance was significantly higher in cancer compared to adjacent normal tissue (*p*_adj_ = 0.040, paired Wilcoxon signed-rank test with Bonferroni correction; Supplementary Data 6). However, the other three species were detected in at least one tissue sample. *F. vincentii* was present in four cancer tissues and two normal tissues, while *F. polymorphum* was detected in four cancer tissues and three normal tissues (Figure 5; Supplementary Data 7). The complete species-level relative abundance data for all detected taxa across all clinical samples are provided in Supplementary Data 8.

### Discussion

The present study demonstrates that full-length 16S rRNA sequencing using Oxford Nanopore technology enables reliable species-level resolution of four clinically significant *Fusobacterium* species and is potentially extendable to the entire genus. This level of discrimination has, to our knowledge, not been achieved using short-read amplicon sequencing of hypervariable regions such as V3-V4. Accurately distinguishing between *F. nucleatum*, *F. animalis*, *F. vincentii* and *F. polymorphum* is essential, as these closely related species differ in CRC prevalence and potential pathogenicity.^17^ Comprehensive taxonomic analyses conclude that previous subspecies of *F. nucleatum sensu lato* should be viewed as individual species.^21^^,^^32^ Recent studies consistently identify *F. animalis* as the species most enriched in CRC samples,^16^^,^^17^ underscoring the need for methods capable of resolving these species.

In addition to their connection with colorectal cancer, these *Fusobacterium* species are also linked to periodontal disease and other infections. Studies that previously separated these bacteria into subspecies have shown that each subspecies can have unique associations with different clinical conditions.^20^ Historically, biological characteristics and disease associations have been attributed broadly to *F. nucleatum sensu lato* without distinguishing individual species.^20^^,^^33^ However, these taxa should be considered as distinct species with potentially different virulence traits and ecological niches, and treating them as functionally interchangeable risks an inaccurate interpretation of CRC associations.^21^^,^^32^ Overlooking phenotypic and genotypic diversity can lead to inaccurate interpretations of CRC associations and pathogenesis and may similarly confound interpretation in other microbiome-associated research contexts.^32^

Although the 16S rRNA gene has been considered insufficient for classifying taxa within the *F. nucleatum* group (*sensu lato*),^10^^,^^20^ and alternative markers such as the *β* subunit of bacterial RNA polymerase *rpoB* and the zinc protease gene have instead been used for subspecies discrimination,^34^ our results demonstrate that this limitation pertains to partial sequences rather than the full-length gene. While alternative markers (e.g., *rpoB*) offer greater sequence variability, the 16S rRNA gene’s conserved primer-binding regions enable broad-range amplification across phyla,^26^^,^^35^^,^^36^ making it more practical for community profiling.

Despite high inter-species ANI values (Figure 3a and 3b), consistent with previous findings,^10^ all four *Fusobacterium* species (e.g., *F. nucleatum*, *F. animalis*, *F. vincentii* and *F. polymorphum*) formed distinct clusters when full-length sequences were used. The Genus-wide phylogenetic analysis confirms that distinct species-level clades are maintained across *Fusobacterium* when full-length 16S rRNA sequences are used (Figure S3). Nevertheless, when the ANI between species is high, discriminatory power may be insufficient,^32^ as illustrated by the *F. periodonticum*/*F. pseudoperiodonticum* pair as visualized in the phylogenetic tree (Figure S3) and in our pure culture experiments (Table 1). Accurate classification also depends on well-curated reference databases. GTDB classifies 60–74% of amplicon sequence variants at the species level compared to <10% with SILVA,^28^ owing to its ANI-based curation and removal of polyphyletic groups.^21^ However, the adoption of updated GTDB nomenclature creates challenges, as “*F. nucleatum*” now refers both to the broad historical group (*sensu lato*) and a single distinct species (*sensu stricto*)^21^.

Several alternative sequencing strategies could have been considered for this application. PacBio HiFi achieves high per-read accuracy, surpassing Nanopore’s accuracy. However, PacBio is primarily available through service providers, limiting accessibility for resource-constrained laboratories. Shotgun metagenomics enables species and strain level resolution but is impractical for tumor samples where microbial DNA comprises < 1% of total reads.^37^ Our Nanopore-based strategy provides a cost-effective and accessible in-house alternative, with an estimated per-sample cost of approximately €7–10 when multiplexing 192 samples. This makes the approach suitable for implementation in routine clinical laboratory settings. The use of a high-fidelity polymerase further reduces off-target amplification in samples with high host-to-microbial DNA ratios,^36^ lowering the number of sequencing reads needed per sample.

To evaluate analytical performance and quantitative accuracy, we assessed the workflow using controlled mock communities. Taxa below 0.066% relative abundance were not reliably detected, although clinically significant *Fusobacterium* species in CRC are typically reported at 0.1–2%,^16^ well within our detection range. The discrepancies between theoretical and observed abundances in the CFU-based suspension (Figure 4b) likely reflect difficulties in bacterial quantification by CFU or differences in DNA extraction efficiency, as DNA concentration-based pooling closely matched expected values (Figure 4c).

Beyond these experimental variabilities, methodological limitations should also be acknowledged. We did not normalize for 16S rRNA gene copy number, as theoretically inferred values may introduce additional bias.^38^ However, we note that the four *Fusobacterium* species have about 5 CNV of the 16S rRNA gene (Figure 3d). Consequently, CNV is unlikely to explain discrepancies between observed and theoretical abundances. In addition, PCR amplification bias may distort abundance estimates, but the high consistency across independent PCR and sequencing runs supports the method’s reproducibility (Figure 2). Another limitation is that this study was conducted in a single-center setting, which may limit generalizability. The clinical cohort (*n* = 20) was deliberately designed as a proof-of-concept dataset to demonstrate technical feasibility. Given the small sample size, the paired Wilcoxon test has limited statistical power for moderate effect sizes, and the clinical comparison should be regarded as exploratory. Confirmation in larger, multi-center cohorts is needed to establish definitive species-level associations with CRC.

Even though the present study was not designed to establish definitive species-level associations with CRC phenotypes or clinical outcomes, we found that *F. animalis* was the most prevalent and had the highest abundance within the four *Fusobacterium* species (Figure 5). Our results are coherent with recent studies, outlined in a review by Jiang *et. al.,* ^39^, which suggests that *F. animalis* may harbor “specialized oncogenic adaptation” compared to *F. nucleatum sensu stricto* and other *Fusobacterium* species. Prior taxonomic revisions, *F. animalis* would have been grouped under *F. nucleatum,* leading to overgeneralization and obscuring its individual contribution to CRC.

An increasing body of evidence suggests that tumorigenic features can differ among closely related bacterial species, making accurate and accessible methods a necessity. The putative tumorigenic potential of specific *Fusobacterium* species highlights the importance of reliable techniques for species-level delineation. By enabling specific identification of the taxa most relevant to CRC, we anticipate that our method will serve as a valuable tool contributing to a deeper understanding of cancer progression and host–microbe interactions in the scope of colorectal cancer. Our full-length 16S sequencing approach, and the implementation of nanoMux demultiplexing tool provides a practical and cost-effective method for species-level *Fusobacterium* profiling. Its accuracy depends on the high-quality and updated reference databases, which are still being refined for certain *Fusobacterium* taxa. By enabling specific identification of the taxa most relevant to CRC, we anticipate that this method will contribute to a deeper understanding of species-specific roles of *Fusobacterium* in cancer progression and host–microbe interactions.

### Materials and methods

#### Fresh frozen patient specimens

As a proof-of-concept for method validation, 20 frozen CRC tumor tissues from the Uppsala–Umeå Comprehensive Cancer Consortium (U-CAN) project^40^ were included in this study. The Swedish Ethical Review Authority (Dnr 2025-01805-01) approved all parts of this project. All patients provided written informed consent.

#### Bacterial strains

*Fusobacterium nucleatum* DSM 15643, *Fusobacterium animalis* DSM 19679, *Fusobacterium polymorphum* DSM 20482, *Fusobacterium vincentii* DSM 19507, *Fusobacterium varium* DSM 19868, *Fusobacterium necrophorum* subsp. *funduliforme* DSM 19678, and *Fusobacterium periodonticum* DSM 19545 were purchased from the Leibniz Institute DSMZ-German Collection of Microorganisms and Cell Cultures GmbH (DSMZ). This study focuses on a subset of these species (*F. animalis*, *F. nucleatum*, *F. vincentii* and *F. polymorphum*) which, when analyzed together, are referred to as *Fuso4* in the present work (Figure 1). All species were grown on Fastidious Anaerobic Agar supplemented with 5% of horse blood (Håtunalab AB). Subsequently, one colony was inoculated into modified Gifu Anaerobic Broth (HyServe) and grown for 48 hours. All incubations were performed in anaerobic conditions in an anoxic workstation (Whitley A35 Anaerobic Workstation, Don Whitley Scientific) at the Umeå Hypoxia Research Facility, Umeå University. For each species, a volume of 10 mL of overnight culture was pelleted by centrifugation for 20 min at 4000 rpm at 4 °C and stored at −20 °C for DNA extraction.

#### Cell lines

Colorectal cancer cell lines RKO (CRL-2577, ATCC) and HT29 (HTB-38, ATCC) were cultured in DMEM high glucose GlutaMAX (Gibco) supplemented with 10% fetal bovine serum (Gibco), 1% penicillin-streptomycin (Thermo Fisher Scientific), 1% MEM Non-Essential Amino Acids Solution (Thermo Fisher Scientific). Cultures were maintained at 37 °C in a humidified incubator with 5% CO_2_ until reaching confluence.

#### In vitro microbial–host models

A mock community composed of human colorectal carcinoma cells and the ZymoBIOMICS Gut Microbial Community Standard (Zymo Research Corporation) was prepared to simulate a gut bacterial sample with a high human cell background. To achieve a 10:1 human to bacterial cell ratio (estimated ratio in a human intestinal biopsy,^37^) 10^6^ RKO cells were mixed with 10^5^ bacterial cells from the ZymoBIOMICS Gut Microbial Community Standard (Supplementary Table 1; Supplementary data 1). The mock community was frozen at −80 °C until sequencing.

Suspensions of host cell/*Fuso4* were prepared by mixing 10^7^ (ratio 1:1) or 10^6^ (ratio 10:1) CFU of *Fuso4* (ratio 1:1:1:1 per species) with 10^7^ HT29 cells (Supplementary Table 1). DNA extraction from the suspensions was performed immediately.

#### DNA extraction

Fresh frozen tissue samples (2–3 mm cube) were homogenized using a Precellys 24 homogenizer (Bertin Technologies) with 1.4 mm ceramic beads, as described previously.^41^ DNA from the homogenate was extracted using the AllPrep DNA/RNA/miRNA Universal kit (Qiagen) according to the manufacturer’s instructions. DNA from the mock community was prepared following the same method.

Total DNA from the individual pellets of *F. animalis*, *F. nucleatum*, *F. vincentii* and *F. polymorphum* and from the HT29/*Fuso4* cell suspensions was extracted using the DNeasy Blood & Tissue Kit (Qiagen) according to the manufacturer’s instructions. Prior to extraction, the HT29/*Fuso4* suspensions were homogenized using NucleoSpin bead type B (Macherey-Nagel) as previously described.^42^ Prior to sequencing, DNA from the *F. animalis*, *F. nucleatum*, *F. vincentii* and *F. polymorphum* cultures was combined into a DNA mixture with a defined proportion for each species. Following DNA extraction, the concentration for all samples was measured using a Qubit 4 fluorometer (Thermo Fisher Scientific) with the Qubit 1x dsDNA HS Assay Kit (Thermo Fisher Scientific).

### 16S rRNA gene amplification, library preparation and nanopore sequencing

To enable multiplexing of 192 uniquely dual-barcoded samples, we used barcoded 16S rRNA primers for 16S amplification, described in the following protocol from PacBio (Procedure & checklist—Amplification of bacterial full-length 16S rRNA gene with barcoded primers). All primers were purchased from LGC Biosearch Technologies with a 5’-Phos modification. Eight 16S rRNA barcoded degenerate forward primers and 24 16S rRNA barcoded degenerate reverse primers were used to amplify the full-length 16S rRNA bacterial genes (v1–v9 regions). The full list of primers used is provided in Supplementary Data 9. The concentration of all primers was 2.5 µM. The PCR amplification and barcoding solution was prepared using 10 µL of Platinum SuperFi II 2X mastermix (Invitrogen), 20 ng template DNA, 2 µL of forward primer, 2 µL of reverse primer and nuclease-free water to a total volume of 20 µL. The reactions were placed in a thermal cycler, where an initial denaturation at 98 °C was followed by 30 cycles of denaturation (10 s at 98 °C), annealing (10 s at 60 °C) and extension (60 s at 72 °C).

DNA quality and amplicon size distributions for all PCR reactions were evaluated using a Tapestation 4200 (Agilent) with Genomic DNA ScreenTape and reagents (Agilent). DNA concentrations were measured using a Synergy LX (Agilent) plate reader with the Qubit 1x dsDNA HS Assay Kit (Thermo Fisher Scientific).

A total of 192 unique barcoded 16S rRNA gene samples were pooled in equimolar amounts and 100 fmol was used as input for library preparation, using the ligation sequencing kit V14 (SQK-LSK114, Oxford Nanopore sequencing), according to the manufacturer’s instructions. In short, end repair and dA-tailing was performed on the pooled samples using NEBNext Ultra II End Repair/dA-tailing Module (NEB), followed by cleaning using AMPure XP beads. The NEBNext Blunt/TA Master Mix (NEB) was added to the mixture, and sequencing adapters were ligated using NEBNext Companion Module v2 for Oxford Nanopore Technologies (NEB). The final sequencing library was cleaned and concentrated using AMPure XP beads and the concentration was measured using a Qubit 4 fluorometer (Thermo Fisher Scientific) with the Qubit 1x dsDNA HS Assay Kit (Thermo Fisher Scientific).

The sequencing library was sequenced using a P2-solo device (Oxford Nanopore sequencing) on a R10.4.1 flow cell (FLO-PRO114M, Oxford Nanopore sequencing). To start the sequencing, MinKNOW (v. 22.08.4) was used, and the sequencing was run until enough reads were reached.

The raw pod5 signal data files generated by MinKNOW were basecalled using Dorado (v. 0.9.0) and the sup basecalling model.

#### Development of nanomux for demultiplexing of noisy dual barcoded reads

To allow for demultiplexing of noisy sequencing reads from Nanopore technologies, we implemented a fuzzy matching approach based on Levenshtein distance and a dynamic programming table. The algorithm searches for approximate matches of known barcode sequences within the ends of each read, allowing up to k insertions, deletions or substitutions. This enables a higher proportion of reads with sequencing errors to still be correctly assigned to the corresponding sample. Instead of requiring unique barcodes for every sample, this method allows for the reuse of barcodes, which enables a greater sample multiplexing with a limited number of forward and reverse barcodes. All tools developed for handling Nanopore reads are implemented in a suite of command-line interface tools named nanoSweet, available at: https://github.com/willros/nanoSweet.

#### Bioinformatic processing and analysis

Quality filtering and read length trimming of the fastq files generated by Dorado were performed using nanotrim from nanoSweet with a minimum length set to 1200 bp and a maximum length set to 2000 bp. The minimum quality was set to a PHRED score of 15.

The surviving reads were demultiplexed using nanoMux from nanoSweet. The number of mismatches was set to 3, scanning was set to 300 and the trimming option was enabled. Before read classification, the GTDB 16S rRNA reference database was filtered to improve taxonomic accuracy and ensure nomenclatural consistency. The filtering process retained only sequences that were unique within the database, removed those containing ambiguous nucleotides (*N*, Y, W, V, S, R, M, K, H, D, B), and ensured each species was represented by at least three reference sequences to provide robust classification. These stringent filtering criteria were applied to maximize the accuracy and reliability of taxonomic assignments. The GTDB filtering script is available at: https://github.com/willros/fusobacterium-ONT-sequencing. Species names were standardized to title case and required a minimum length of eight characters to maintain proper nomenclatural formatting.

A custom bioinformatic pipeline using Snakemake implemented in Python was applied to analyze the demultiplexed fastq. In short, the reads were deduplicated using seqkit (v. 2.0.0, https://github.com/shenwei356/seqkit) and chimeras were removed using vsearch (v. 2.29.4, https://github.com/torognes/vsearch). The usearch function from vsearch was used to classify each read, using the filtered GTDB file.

Due to inconsistencies between NCBI and GTDB, some species in the ZymoBIOMICS Gut Microbiome Standard are labeled differently (for example, *F. nucleatum* in NCBI corresponds to *F. animalis* in GTDB, and *Lactobacillus fermentum* in NCBI corresponds to *Limosilactobacillus fermentum* in GTDB). In this study, the GTDB nomenclature was used for all analyses.

All remaining sequences from the four *Fusobacterium* species were then used as input for phylogenetic analysis and average ANI estimation. After removing ambiguous sequences (clusters “J” and “D”; see Results), the final dataset included 7, 54, 31, and 34 sequences for *F. nucleatum*, *F. polymorphum*, *F. vincentii*, and *F. animalis*, respectively. Sequences were aligned using MUSCLE (v5.3). A maximum likelihood phylogenetic tree was constructed using iq-tree (v3.0.1) with default settings. The resulting tree was visualized as an unrooted phylogram using the R library ggtree (v3.12.0). Pairwise ANI values were calculated using skani (v0.3.0) in triangle mode with the arguments --full-matrix and --small-genomes. Although skani is primarily designed for genome-level comparisons, the --small-genomes option enables reliable ANI estimation for short sequences such as single genes. The resulting ANI values were consistent with traditional alignment-based identity calculations from MUSCLE, confirming the validity of this approach for 16S rRNA gene sequences. To assess whether the discriminatory power of the full-length 16S rRNA sequence extends to the broader *Fusobacterium* genus, we conducted the same analysis with a total of 215 sequences of the 16S rRNA gene, corresponding to 13 species (Supplementary Data 10). Two sequences were excluded for the phylogenetic tree visualization due to excessive divergence relative to all other sequences: one *F. varium* (RS_GCF_015555635.1) and one *F. ulcerans* (RS_GCF_938039905.1), resulting in a final dataset of 213 sequences.

All subsequent analyses and visualizations were performed in R (v4.2) and Python (v3.11). In R, data wrangling and visualization were performed using the tidyverse packages (v. 2.0.0). For Python, data curation and figure generation were performed using Seaborn (v0.13), Polars (v1.6), BioPython (v. 1.85) and Matplotlib (v3.9). The rrnDB (v5.10)^43^ was used to estimate 16S rRNA gene CNV for each *Fusobacterium* species. The 16S rRNA sequences from GTDB were further used to calculate the mean GC content for each species according to the following formula:

$$\text{GC}\%=\frac{\sum (\text{GC}-\text{content}/\text{sequence length})}{n_{\text{sequences}}}$$

#### Statistical analysis

All statistical analyses were conducted using R (v4.2). The comparison of *Fusobacterium* species abundance between cancer and normal tissue was performed using a paired Wilcoxon signed-rank test for each of the three *Fusobacterium* species detected above the threshold in the clinical cohort (*k* = 3 comparisons: *F. animalis*, *F. vincentii* and *F. polymorphum*; *F. nucleatum* was not detected and therefore not tested). Multiple hypothesis testing correction was performed using the Bonferroni method (adjusted significance threshold *α* = 0, 05/3 ≈ 0,0167). A Bonferroni-corrected *p-*value < 0.05 was considered to indicate statistical significance.

## Supplementary Material

Supplementary data_Gut Microbes Revision 1.xlsx supplementary_data_gut_microbes.xlsx

Supplementary Figures_Gut Microbes Revision 1.pdf supplementary_figures_gut_microbes.pdf

Supplementary Table_Gut Microbes Revision 1.pdf supplementary_table_gut_microbes.pdf

## Data Availability

Raw sequencing data are available at the Zenodo repository (doi: 10.5281/zenodo.17601340). Relative abundance data for the clinical cohort and figures are provided in the supplementary data.

## Code availability

The underlying code and pipelines for this study can be accessed through GitHub (https://github.com/willros/fusobacterium-ONT-sequencing). The novel demultiplexing software nanoMux is also publicly available at GitHub (https://github.com/willros/nanoSweet).
