# Supplementary material for: Full-length 16S rRNA nanopore sequencing enables species resolution of Fusobacterium associated with colorectal cancer
Source: Gut Microbes. 2026 Apr 10;18(1):2656004. doi: 10.1080/19490976.2026.2656004 (PMC13078227; doi:10.1080/19490976.2026.2656004)
Supplement: Supplementary Figures_Gut Microbes Revision 1.pdf — supplementary_figures_gut_microbes.pdf [file KGMI_A_2656004_SM2097.pdf]

Supplementary Figures

Supplementary Figure 1. Demultiplexing performance across mismatch thresholds (k = 0–3) of nanoMux between unique read recovery against multi-mapped reads. Each panel displays the percentage of total reads assigned uniquely to one sample (1) versus reads mapped to multiple samples (2) at each k value. k represents the maximum number of allowed mismatches in barcode matching using Levenshtein distance. A mismatch threshold of k = 3 was selected as optimal balance.

Supplementary Figure 2. Histograms of PHRED-score values from positive controls sequenced with the latest Oxford Nanopore chemistry and flow cells. Histograms of positive controls with number of reads > 10000. Each facet represents a unique sample. All sequences with a Q-value < 15 have been filtered out.

Supplementarty Figure 3. Genus-wide phylogenetic tree of Fusobacterium using full-length 16S rRNA sequence. Unrooted phylogenetic tree constructed from 213 full-length 16S rRNA gene sequences representing all 13 *Fusobacterium* species available in GTDB. Species are color-coded by tip points. This analysis extends the four-species phylogeny shown in Fig. 3C to the full genus. *F. nucleatum* (n = 7), *F. polymorphum* (n = 54), *F. vincentii* (n = 31), and *F. animalis* (n = 34) — are shown alongside nine additional species: *F. hwasookii* (n = 17), *F. necrophorum* (n = 15), *F. varium* (n = 14), *F. pseudoperiodonticum* (n = 14), *F. canifelinum* (n = 9), *F. ulcerans* (n = 7), *F. mortiferum* (n = 5), *F. periodonticum* (n = 4), and *F. necrogenes* (n = 4). Two sequences were excluded prior to tree construction due to excessive divergence from all other sequences: one *F. varium* (RS_GCF_015555635.1) and one *F. ulcerans* (RS_GCF_938039905.1). The tree was constructed using MUSCLE (v5.3) for multiple sequence alignment and IQ-TREE (v3.0.1) with default settings for maximum likelihood inference. Visualization was performed using ggtree (v3.12.0) in R with an unrooted layout.

Fig. S1

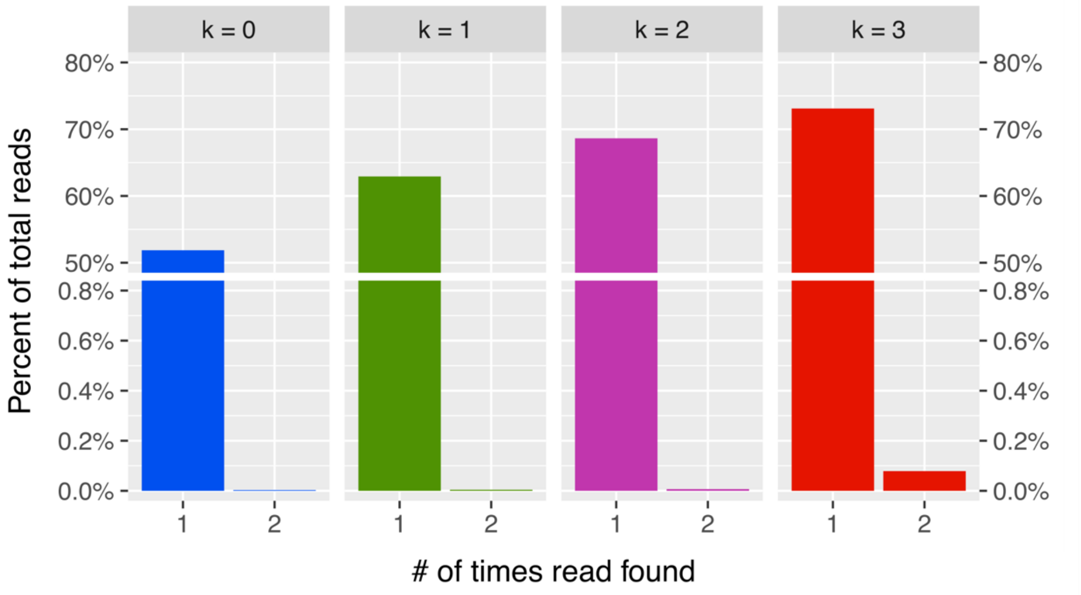

k = 0
k = 1
k = 2
k = 3
80%
70%
60%
50%
0.8%
0.6%
0.4%
0.2%
0.0%
1
2
Percent of total reads
# of times read found

Fig. S2

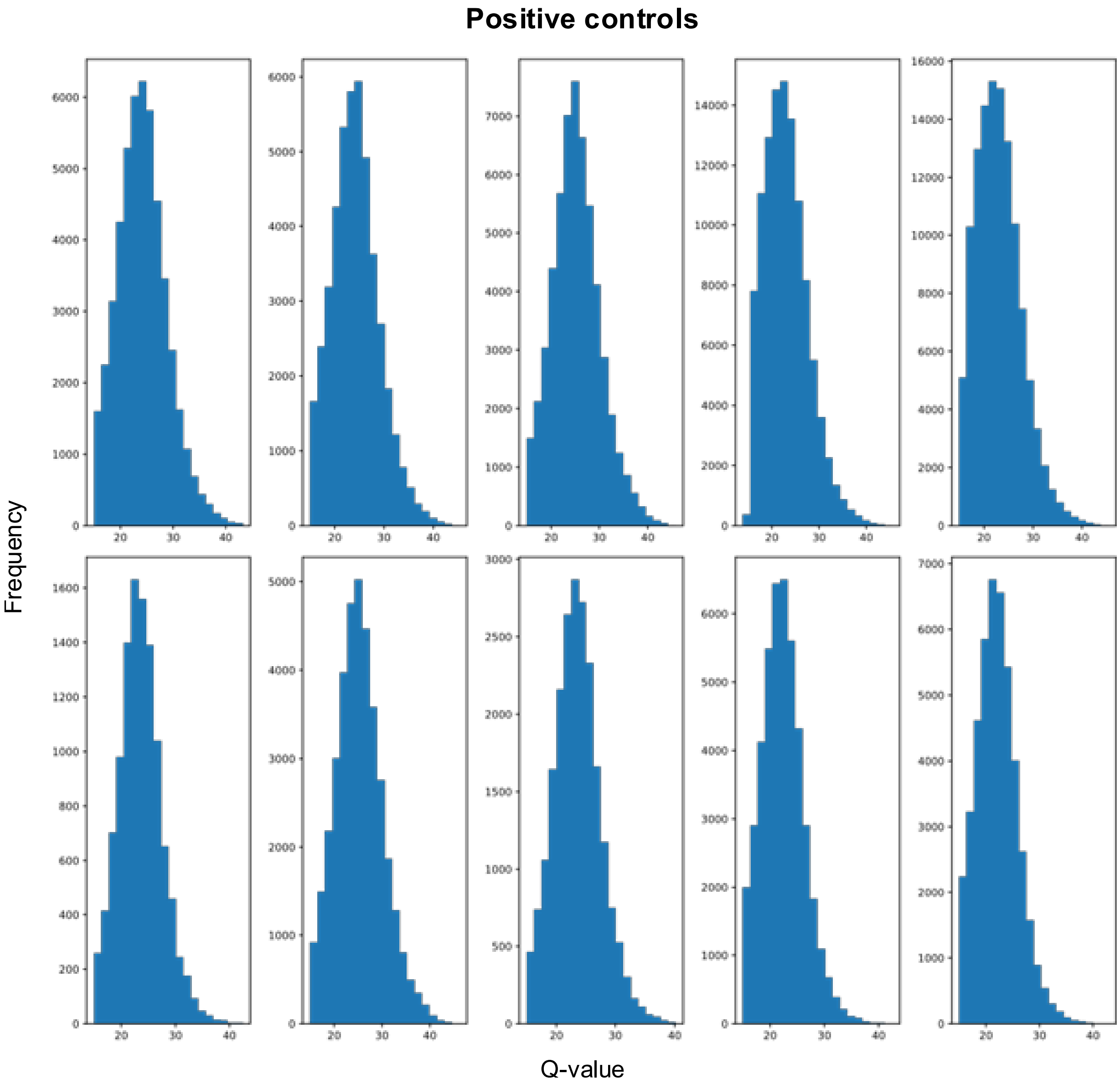

Fig. S3

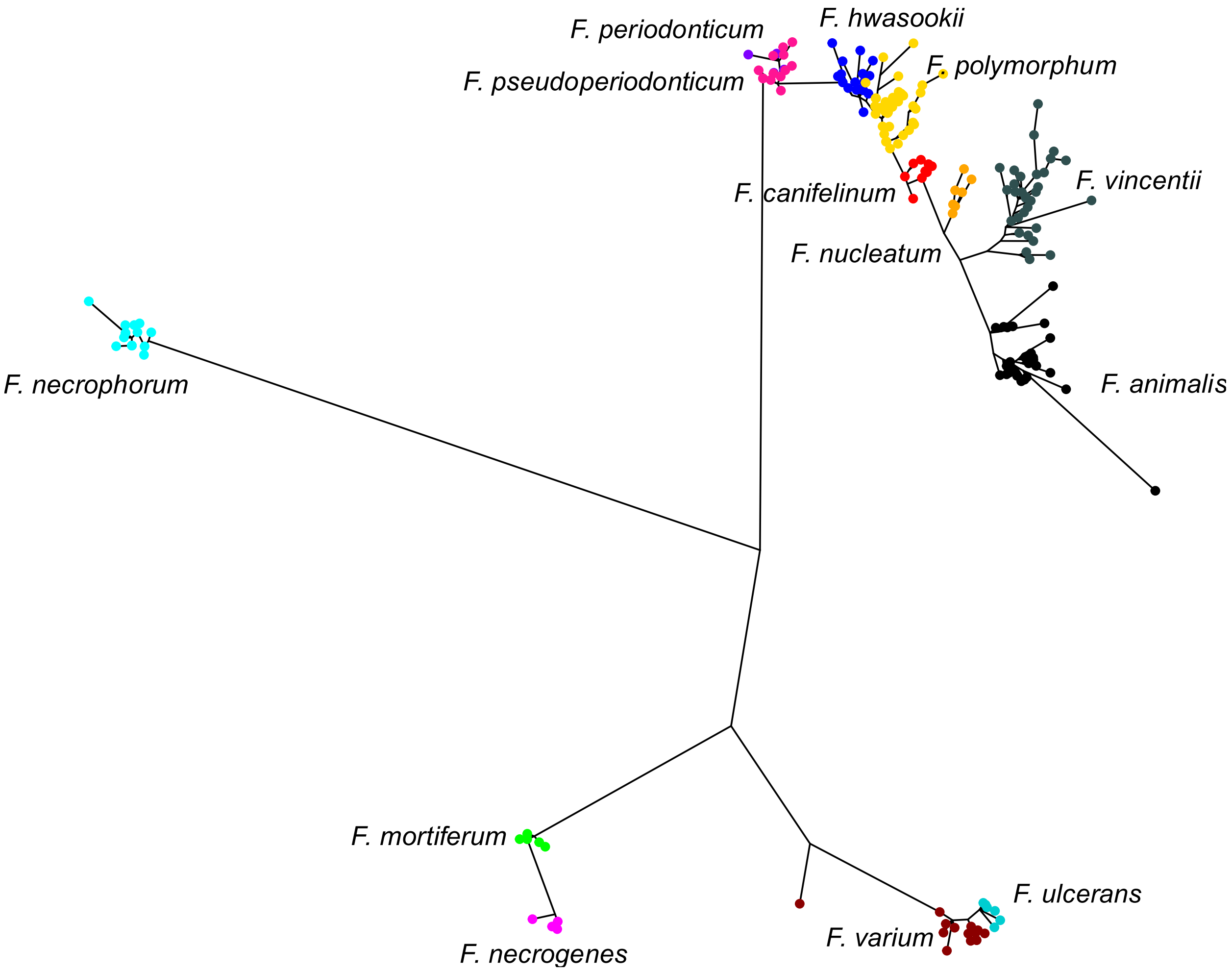
F. periodonticum
F. hwasookii
F. polymorphum
F. pseudoperiodonticum
F. canifelinum
F. vincentii
F. nucleatum
F. necrophorum
F. animalis
F. mortiferum
F. ulcerans
F. varium
F. necrogenes
