# Supplementary material for: Full-length 16S rRNA nanopore sequencing enables species resolution of Fusobacterium associated with colorectal cancer
Source: Gut Microbes. 2026 Apr 10;18(1):2656004. doi: 10.1080/19490976.2026.2656004 (PMC13078227; doi:10.1080/19490976.2026.2656004)
Supplement: Supplementary Table_Gut Microbes Revision 1.pdf — supplementary_table_gut_microbes.pdf [file KGMI_A_2656004_SM2098.pdf]

Supplementary table 1. Overview of experimental samples and input material used in this study.

Sample name	Material type	Ratio	Input amount in the mix	Note
Mock community	Live bacteria	10:1	10^6 RKO cells<br>10^6 CFU ZymoBIOMICS	Mixed prior to DNA extraction
Fuso4	Live bacteria	1:1:1:1	2.5 x 10^6 CFU *F. nucleatum*<br>2.5 x 10^6 CFU *F. animalis*<br>2.5 x 10^6 CFU *F. vincentii*<br>2.5 x 10^6 CFU *F. polymorphum*	
Host cell / *Fuso4*	Human cells + live bacteria	1:1	10^7 HT29 cells<br>2.5 x 10^6 CFU *F. nucleatum*<br>2.5 x 10^6 CFU *F. animalis*<br>2.5 x 10^6 CFU *F. vincentii*<br>2.5 x 10^6 CFU *F. polymorphum*	Mixed prior to DNA extraction
Host cell / *Fuso4*	Human cells + live bacteria	10:1	10^7 HT29 cells<br>2.5 x 10^5 CFU *F. nucleatum*<br>2.5 x 10^5 CFU *F. animalis*<br>2.5 x 10^5 CFU *F. vincentii*<br>2.5 x 10^5 CFU *F. polymorphum*	Mixed prior to DNA extraction
Fuso4 DNA mix	Extracted DNA	/	3.17 ng *F. nucleatum* DNA<br>2.25 ng *F. animalis* DNA<br>7.38 ng *F. vincentii* DNA<br>4.78 ng *F. polymorphum* DNA	Mixed after individual DNA extraction
